# Changes in Volume of Normal Erythrocytes Incubated with Plasma of Patients with Malignant Tumours

**DOI:** 10.1038/bjc.1956.93

**Published:** 1956-12

**Authors:** Y. M. Bromberg, M. B. Erdreich


### CHANGES IN VOLUME OF NORMAL ERYTHROCYTES INCUBATED WITH PLASMA OF PATIENTS WITH MALIGNANT TUMOURS

Y. M. BROMBERG AND M. B. ERDREICH

*From the Gynaecologic and Obstetric Department and Clinical Research Laboratory, Hadassah University Hospital, Jerusalem, Israel*

Received for publication October 15, 1956

INTRAVASCULAR haemolysis may occur in malignant neoplastic diseases and is sometimes responsible for anaemia observed in these patients (Stats, Rosenthal and Wasserman, 1947; Hyman, 1954). This observation led us to study the haemolytic activity of plasma of patients with malignant tumours. The preliminary results indicate that although frank haemolysis does not result from incubation of normal erythrocytes with the plasma of these patients, the mean corpuscular volume (MCV) of the incubated red cells is usually increased significantly. No such effect was obtained with plasma of normal individuals, or of patients with benign tumours.

### MATERIAL

(1) *Control series*

(a) *Normal controls.*—The plasma of 28 men and women from 25 to 55 years of age was studied. All of these healthy subjects were members of the hospital staff with normal blood count, urinalysis, blood pressure, X-ray of the chest and no gross abnormalities on physical examination.

(b) *Patients with benign tumours.*—This series consisted of 8 patients with the following histologically confirmed benign tumours: Fibro-adenoma of the breast, 1 case; hydatidiform mole, 2 cases; myoma of the uterus, 2 cases; papilloma of the bladder, 1 case; serous cystadenoma of the ovary, 2 cases.

(2) *Patients with malignant tumours*

This series consisted of 15 patients suffering from the following malignant tumours: adenocarcinoma of the breast, 3 cases; granulosa cell carcinoma of the ovary, 2 cases; and 1 case each of: squamous cell carcinoma of the uterine cervix, squamous cell carcinoma of the penis, adenocarcinoma of the endometrium, adenocarcinoma of the ovary, sarcoma of the uterus, serous cystadenocarcinoma of the ovary, papillary transitional carcinoma of the bladder, adenocarcinoma of the colon, generalized carcinomatosis of the peritoneum and mucoid carcinoma of the lung.

### METHOD

Blood samples of 10 ml. were taken from the cubital vein and added to 0·05 ml. of a solution of sodium heparin containing 1000 Toronto units per ml. The MCV of erythrocytes was determined from the red cell count and haematocrit reading. The erythrocytes counts were averaged in two Spencer counting chambers.

The haematocrit was determined in Wintrobe tubes centrifuged for 45 minutes at 3000 r.p.m. in an international centrifuge.

The erythrocytes were separated from the plasma in a specially constructed siliconized centrifuge tube with a constricted portion, to facilitate accurate filling to the 4 ml. mark (Fig. 1). After being filled with blood, the tube was centrifuged

FIG. 1.—Special tube used in incubating blood phases. Overall length 90 mm. Constricted portion allows for accurate filling to 4·0 ml. mark.

at 1500 r.p.m. for 5 minutes. The plasma was then removed and the plasma of another subject of the same blood group added, to bring the total volume again to the 4 ml. mark. The erythrocytes were then resuspended by repeated gentle inversion and the system incubated at 37° C., for one hour. The tube was then again repeatedly inverted and a sample taken for determining the haematocrit. The MCV after incubation was determined by relating this second haematocrit reading to the initial count. Thus repeated erythrocytes counts which might introduce a high degree of error, were obviated.

### RESULTS

Neither normal plasma, nor the plasma of patients with benign tumours modified the MCV of normal erythrocytes (Tables I and II). Conversely, normal plasma had no effect on the erythrocytes of patients with benign tumours (Table III).

On the other hand, the plasma of 11 out of 15 patients with malignant tumours significantly increased the MCV of normal erythrocytes (Table IV).

The degree of erythrocytes enlargement did not depend on the extension of the malignant disease, nor the degree of deterioration of the patient's general condition. In fact, the greatest increase in MCV of normal erythrocytes was produced by the plasma of patients with squamous cell carcinoma of the cervix and squamous cell carcinoma of the penis without evidence of metastases and in fair general condition (Table IV, case 1, 2). On the other hand, no change in the MCV of normal erythrocytes occurred in the plasma of a patient in the terminal stage of adenocarcinoma of the colon (Table IV, case 14).

TABLE I.—*The Effect of Normal Plasma on the Mean Corpuscular Volume (MCV) of Erythrocytes of Other Normal Persons in Incubated Systems*

| | Case | | | | | | | | | |
|---|---|---|---|---|---|---|---|---|---|---|
| | 1 | 2 | 3 | 4 | 5 | 6 | 7 | 8 | 9 | 10 |
| MCV before incubation | 78·25 | 95·18 | 86·31 | 85·00 | 85·71 | 88·42 | 86·91 | 79·79 | 87·77 | 90·00 |
| MCV after incubation | 78·04 | 96·40 | 86·31 | 85·00 | 86·73 | 89·48 | 85·93 | 80·80 | 88·88 | 90·00 |
| Difference . . . | −0·19 | +1·22 | 0·00 | 0·00 | +1·02 | +1·06 | −0·92 | +1·01 | +1·11 | 0·00 |

| | Case | | | | | | | | | |
|---|---|---|---|---|---|---|---|---|---|---|
| | 11 | 12 | 13 | 14 | 15 | 16 | 17 | 18 | 19 | 20 |
| MCV before incubation | 80·00 | 90·22 | 78·90 | 83·87 | 83·14 | 89·91 | 92·68 | 86·00 | 85·99 | 89·37 |
| MCV after incubation | 78·94 | 90·22 | 81·88 | 84·95 | 83·14 | 88·10 | 90·24 | 85·00 | 88·11 | 89·37 |
| Difference . . . | −1·06 | 0·00 | +2·98 | +1·08 | 0·00 | −1·81 | −2·44 | −1·00 | +2·12 | 0·00 |

| | Case | | | | | | | |
|---|---|---|---|---|---|---|---|---|
| | 21 | 22 | 23 | 24 | 25 | 26 | 27 | 28 |
| MCV before incubation | 88·42 | 83·67 | 91·11 | 94·30 | 82·37 | 80·91 | 83·51 | 89·28 |
| MCV after incubation | 88·42 | 82·66 | 91·11 | 94·30 | 82·37 | 78·84 | 83·51 | 88·17 |
| Difference . . . | 0·00 | −1·01 | 0·00 | 0·00 | 0·00 | −2·07 | 0·00 | −1·09 |

Mean of difference was −0·82 ;
Standard deviation was ±0·84 ;
Standard error was ±0·16.

TABLE II.—*The Effect of Plasma of Patients with Benign Tumours on the Mean Corpuscular Volume (MCV) of Erythrocytes of Controls in Incubated Systems*

| Case | Age | Histological diagnosis | MCV* before incubation | MCV† after incubation | Difference |
|---|---|---|---|---|---|
| 1 | 72 | Papilloma of the bladder | 86·46 | 88·54 | +2·08 |
| 2 | 46 | Fibro-adenoma of the breast | 82·99 | 82·99 | 0·00 |
| 3 | 25 | Hydatidiform mole | 89·48 | 89·48 | 0·00 |
| 4 | 27 | ,, ,, | 85·50 | 86·00 | +0·50 |
| 5 | 53 | Myoma of the uterus | 91·00 | 90·00 | −1·00 |
| 6 | 49 | ,, ,, ,, ,, | 86·04 | 86·04 | 0·00 |
| 7 | 53 | Serous cystadenoma of the ovary | 95·55 | 95·55 | 0·00 |
| 8 | 38 | ,, ,, ,, ,, ,, | 80·51 | 80·51 | 0·00 |

*† The difference between the two means was 0, 2 ;
The standard error was 1·1.
The level of significance (" T Test ") as related to the normal controls series was 0, 2 (not significant).

TABLE III.—*The Effect of Plasma of Control Persons on the Mean Corpuscular Volume (MCV) of Erythrocytes of Patients with Benign Tumours in Incubated Systems*

| Case* | MCV before incubation | MCV after incubation | Difference† |
|---|---|---|---|
| 1 | 90·74 | 87·85 | −2·89 |
| 2 | 88·04 | 88·04 | 0·00 |
| 3 | 82·66 | 82·66 | 0·00 |
| 4 | 90·00 | 89·00 | −1·00 |
| 5 | 89·04 | 89·04 | 0·00 |
| 6 | 85·00 | 85·00 | 0·00 |
| 7 | 85·50 | 85·50 | 0·00 |
| 8 | 92·04 | 92·04 | 0·00 |

* The number of each case corresponds to the case with the same number in Table II.

† Mean of difference was −0·48 ;
Standard deviation was ±0·85 ;
Standard Error was ±0·32.

The level of significance (" T Test ") as related to the normal controls series was 1,2 (not significant).

TABLE IV.—*The Effect of Plasma of Patients with Malignant Tumours on the MCV of Erythrocytes of Normal Persons in Incubated Systems*

| Case | Age | Histological diagnosis | Metastases | General condition | MCV before incubation | MCV after incubation | Absolute difference |
|---|---|---|---|---|---|---|---|
| 1 | 55 | Squamous carcinoma of cervix | Absent | Fair | 91·13 | 113·30 | +24·63 |
| 2 | 42 | Squamous carcinoma of penis | ,, | ,, | 86·68 | 101·48 | +14·80 |
| 3 | 43 | Generalized carcinoma of peritoneum | Present | Poor | 85·36 | 97·56 | +12·20 |
| 4 | 37 | Adenocarcinoma of the breast | ,, | Fair | 93·00 | 109·41 | +16·41 |
| 5 | 50 | Ditto | ,, | Poor | 91·88 | 97·88 | +6·00 |
| 6 | 48 | Ditto | ,, | ,, | 79·00 | 84·00 | +5·00 |
| 7 | 39 | Granulosa cell carcinoma of ovary | ,, | Fair | 86·21 | 93·75 | +7·54 |
| 8 | 60 | Ditto | ,, | ,, | 82·35 | 87·25 | +4·90 |
| 9 | 37 | Serous papillary cystadenocarcinoma of the ovary | Absent | ,, | 80·93 | 80·93 | 0·00 |
| 10 | 60 | Adenocarcinoma of the ovary | Present | ,, | 90·24 | 90·24 | 0·00 |
| 11 | 66 | Mucoid carcinoma of lung | ,, | ,, | 91·11 | 97·77 | +6·66 |
| 12 | 49 | Adenocarcinoma of endometrium | ,, | Poor | 97·43 | 101·33 | +3·90 |
| 13 | 44 | Sarcoma of the uterus | ,, | ,, | 89·74 | 5·00 | +5·26 |
| 14 | 56 | Adenocarcinoma of colon | ,, | ,, | 88·88 | 88·88 | 0·00 |
| 15 | 77 | Papillary transitional cell carcinoma of bladder | Absent | Fair | 86·00 | 88·00 | +2·00 |

Mean of difference was 7·29 ;
Standard error was ±1·9 ;
Standard deviation was ±6·73.

The level of significance (" T Test ") as related to the normal controls series was 4, 26 (significant.)

When the erythrocytes of patients with malignant disease were incubated with normal plasma, no increase in MCV occurred (Table V).

TABLE V.—*The Effect of Plasma of Control Persons on the Mean Corpuscular Volume (MCV) of Erythrocytes of Patients with Malignant Tumours in Incubated Systems*

| Case* | MCV before incubation | MCV after incubation | Difference† |
|---|---|---|---|
| 1 | 93·02 | 91·08 | −1·94 |
| 2 | 90·45 | 91·71 | 1·26 |
| 3 | 92·64 | 94·44 | 1·80 |
| 4 | 89·48 | 85·01 | −4·47 |
| 5 | 91·88 | 88·65 | −3·23 |
| 6 | 100·00 | 96·66 | −3·34 |
| 7 | 90·44 | 87·85 | −2·59 |
| 8 | 93·54 | 91·93 | −1·61 |
| 9 | 80·00 | 80·00 | 0·00 |
| 10 | 92·68 | 85·36 | −7·32 |
| 11 | 85·59 | 82·35 | −3·24 |
| 12 | 88·37 | 88·37 | 0·00 |
| 13 | 94·44 | 94·44 | 0·00 |
| 14 | 87·30 | 87·30 | 0·00 |
| 15 | 89·48 | 88·42 | −1·06 |

* The number of each case correspond to the case with the same number in Table IV.

† Mean of Difference was −1·64 ;
Standard deviation was ±2·02 ;
Standard error was ±0·7.

The level of significance (" T Test ") as related to the normal controls series was 1, 1 (not significant.)

### DISCUSSION

Increase in the volume of erythrocytes occurs as a first step in the course of the haemolytic process. These prolytic changes in volume develop before complete lysis with hypotonic solutions, saponin and lysolecithin.

The increase in volume of normal erythrocytes on incubation for one hour with the plasma of patients with malignant disease, may also represent a haemolytic phenomenon and suggest that such plasma has increased haemolytic activity.

It has been shown that when normal blood is incubated for prolonged periods of time (12 hours or more), the erythrocytes may undergo prolytic changes in shape with increase in volume (Gillespie, 1943). These changes may be due to the presence of the haemolysins demonstrated in the human plasma incubated at 37° (Bergenhem and Fähraeus, 1936). Incubation is thought to release active haemolysins from inactive complexes with inhibitors (Ponder, 1951). The finding of the haemolytic substances in saline extracts of different tissues suggests possible source of these autohaemolysins.

Furthermore, the haemolytic activity of extracts of malignant tumours is much greater than that of normal tissues (Ponder and Nesmith, 1952). The increase in β glucuronidase and hyaluronidase content of the incubated extracts of malignant tumours may be responsible for their increased haemolytic activity (Leroy and Spurrier, 1955).

It is possible that the property of the plasma of patients with malignant diseases described in this study, may be due to similar enzymatic activity. Our data appear to indicate that in the conditions we use, the effective concentration of plasma lysins in patients with malignant tumours, is significantly higher than in those with benign tumours or in healthy subjects.

Further work is carried out at present in order to investigate the hypothesis.

### SUMMARY

The MCV of normal erythrocytes was significantly increased by incubation with the plasma of 11 out of 15 patients with malignant tumours. The plasma of normal subjects or of patients with benign tumours did not have this effect. It is suggested that the erythrocytes enlargement is prolytic and due to increased haemolytic activity of the plasma of patients with malignant neoplasms.

### REFERENCES

Bergenhem, B. and Fåhraeus, R.—(1936) *Z. ges. exp. Med.*, **97**, 555.
Gillespie, W. A.—(1943) *Quart. J. exp. Physiol.*, **33**, 113.
Hyman, G. A.—(1954) *J. Hemat.*, **9**, 911.
Leroy, E. P. and Spurrier, W.—(1955) *Blood.*, **10**, 912.
Ponder, E.—(1951) *Ibid.*, **6**, 559.
*Idem* and Nesmith, J.—(1952) *Cancer Res.*, **19**, 104.
Stats, D., Rosenthal, N. and Wasserman, L. R.—(1947) *Amer. J. clin. Path.*, **17**, 885.


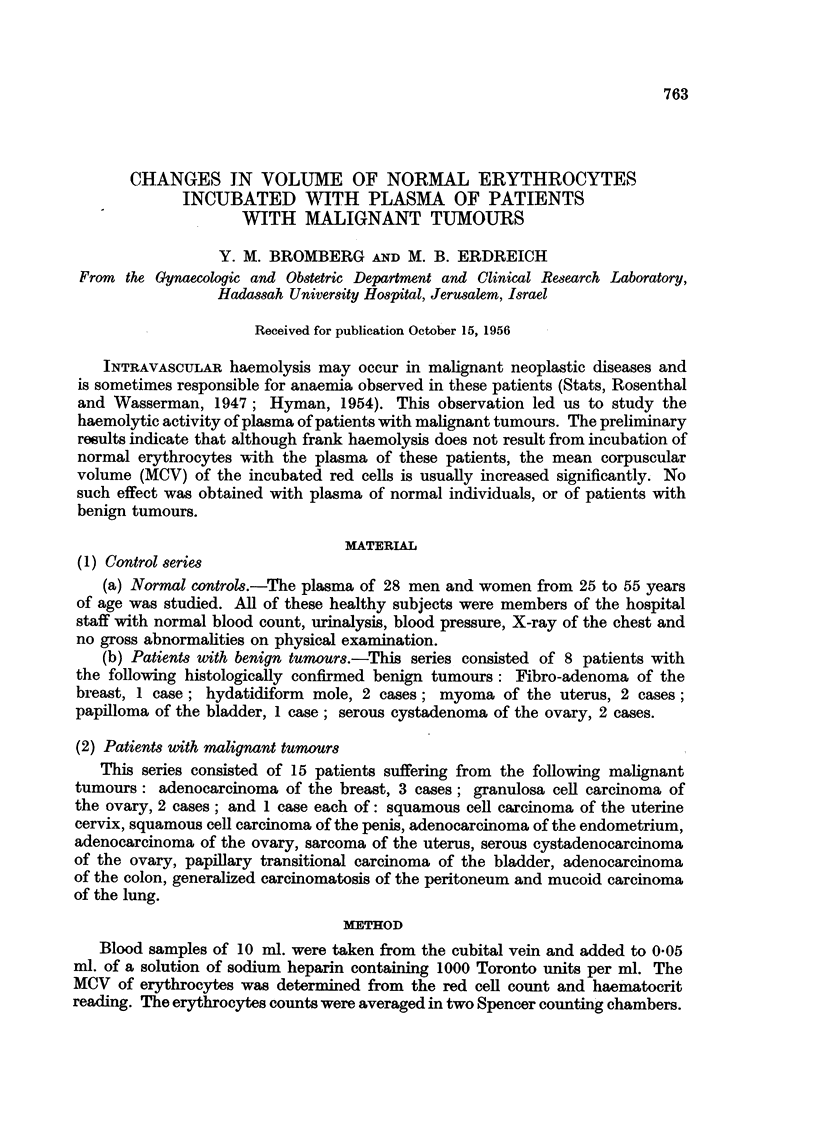

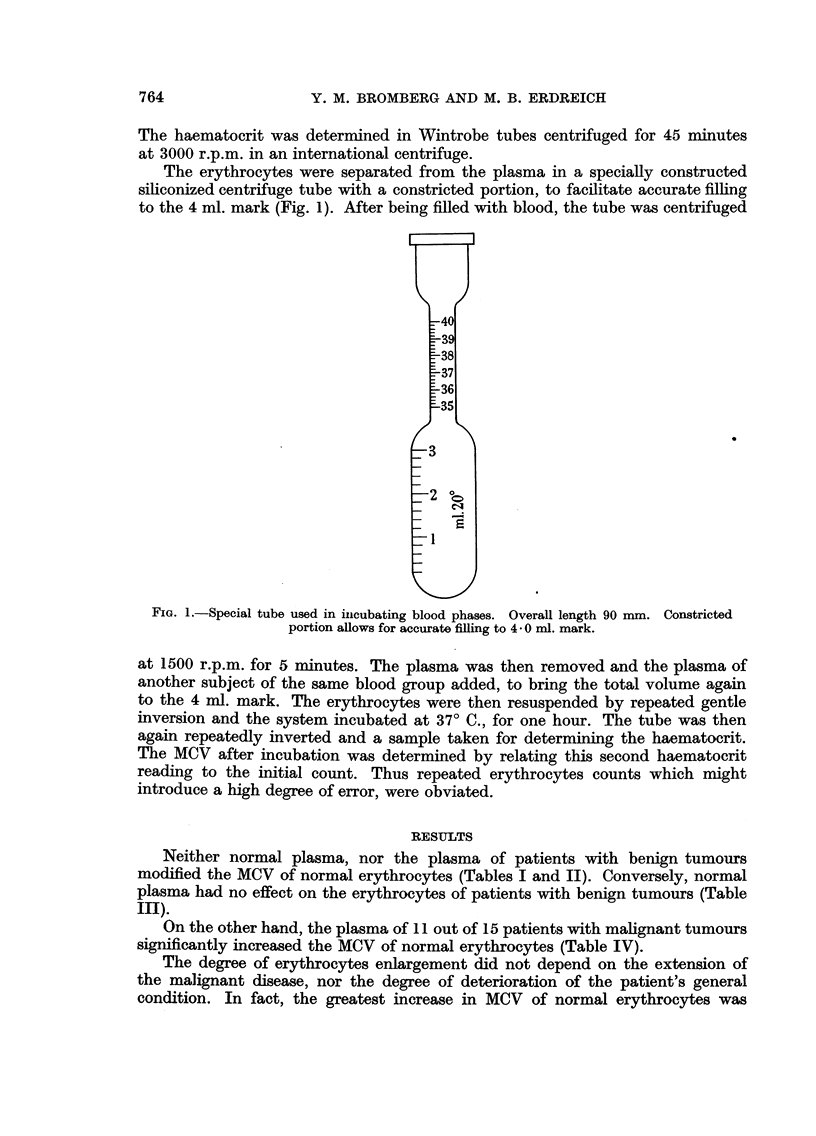

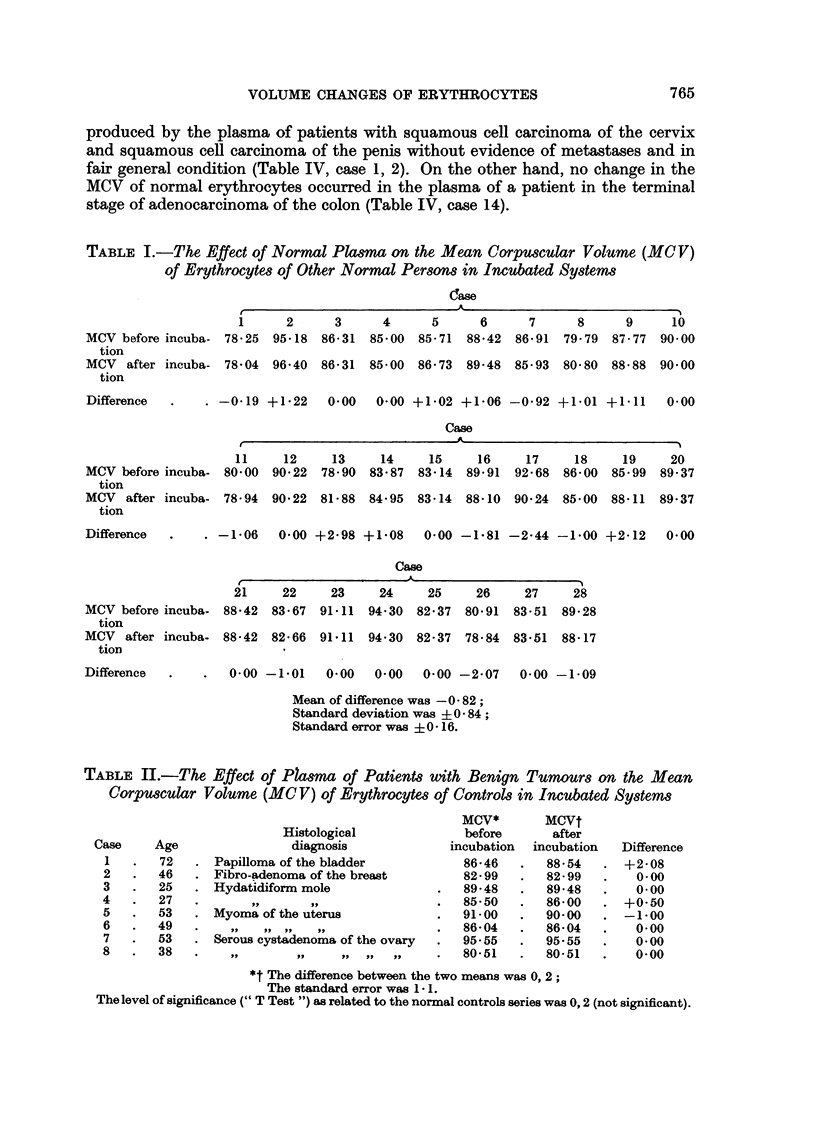

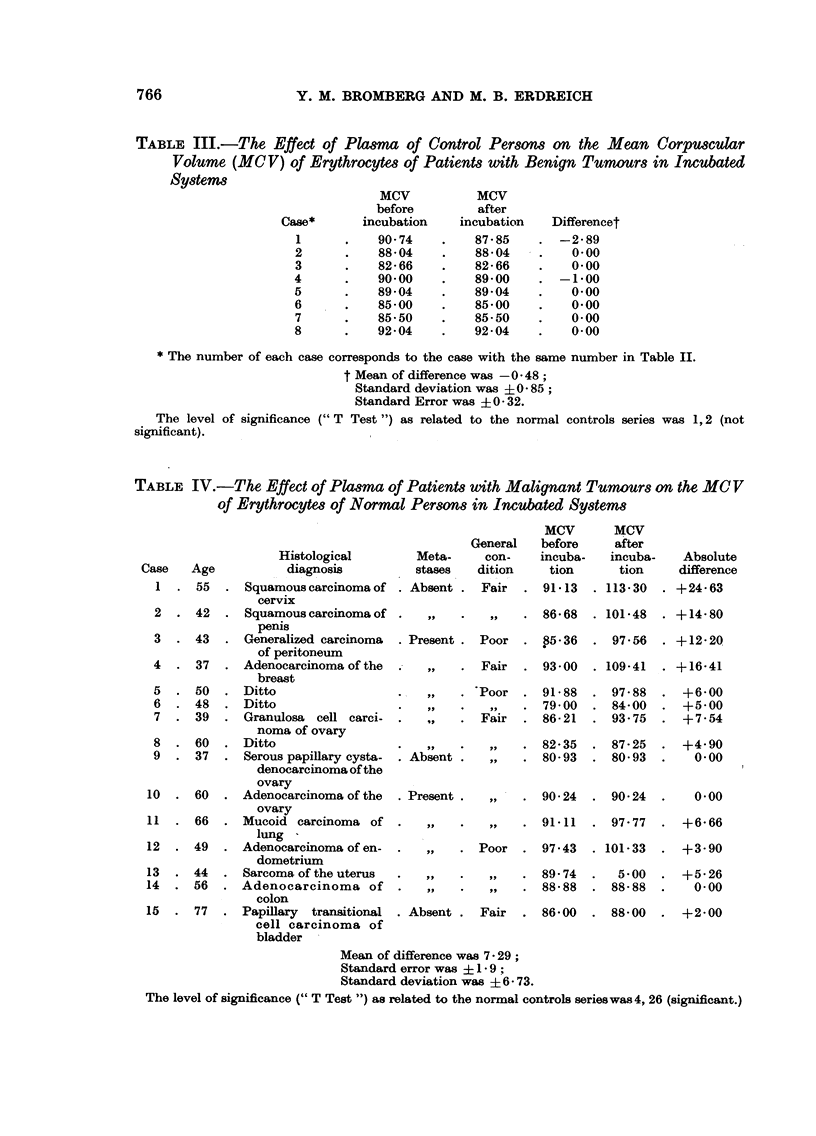

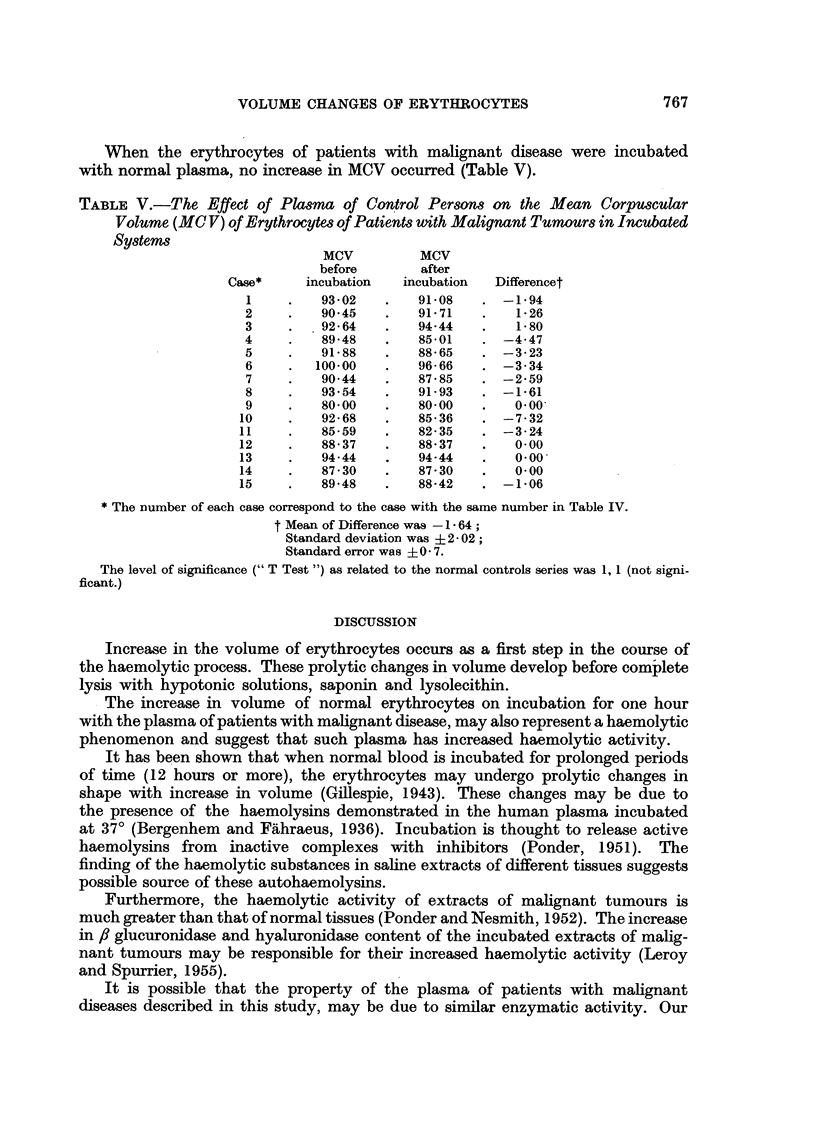

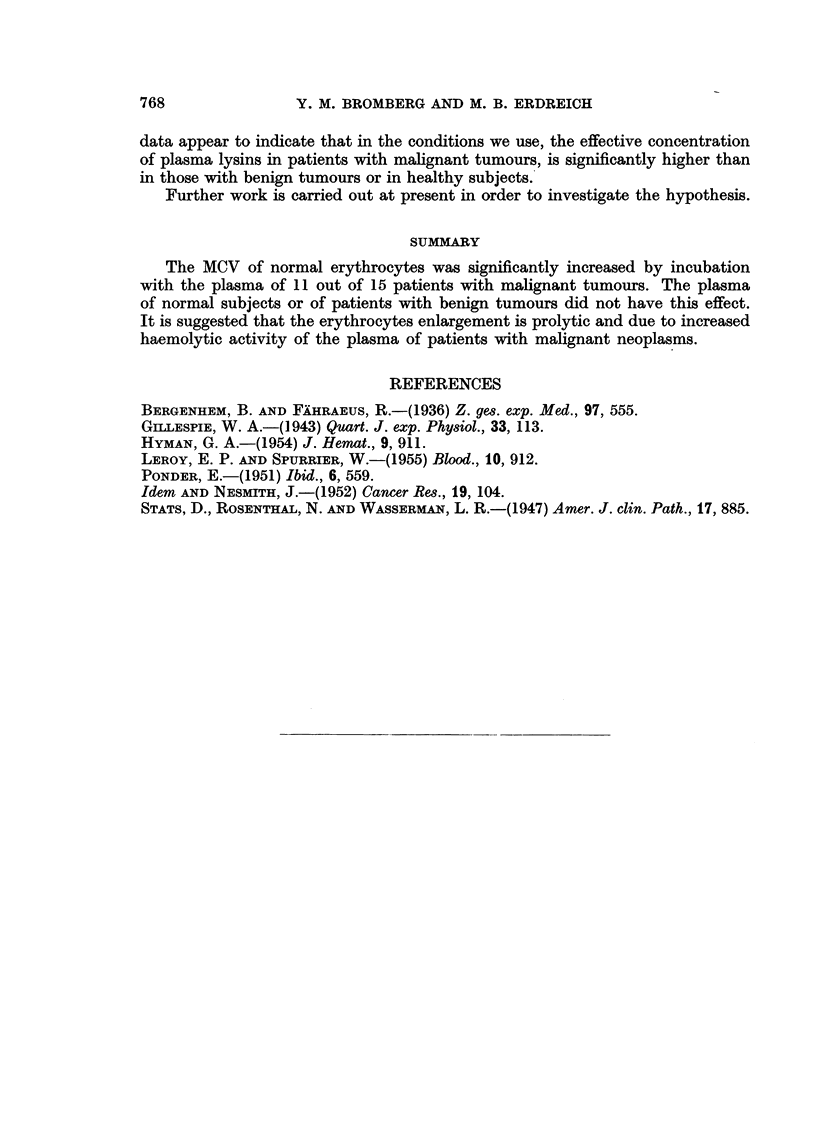


## References

[OCR_00548] HYMAN G. A. (1954). Studies on anemia of disseminated malignant neoplastic disease. I. The hemolytic factor.. Blood.

[OCR_00550] LEROY E. P., SPURRIER W. (1955). Hemolytic property of some carbohydrases; their possible role in red cell destruction.. Blood.

[OCR_00551] PONDER E. (1951). Certain hemolytic mechanism in hemolytic anemia.. Blood.

[OCR_00553] PONDER E., NESMITH J. (1952). Hemolysins in spontaneous mouse breast tumors as compared to those in normal mouse tissue.. Cancer Res.

